# Surgical Treatment with Locoregional Flaps for the Eyelid: A Review

**DOI:** 10.1155/2017/6742537

**Published:** 2017-10-26

**Authors:** Federico Lo Torto, Luigi Losco, Nicoletta Bernardini, Manfredi Greco, Gianluca Scuderi, Diego Ribuffo

**Affiliations:** ^1^Plastic Surgery Unit, Department of Surgery, Policlinico Umberto I Sapienza University of Rome, Rome, Italy; ^2^Dermatology Unit “Daniele Innocenzi”, Department of Medical-Surgical Sciences and Bio-Technologies, Sapienza University of Rome, Polo Pontino, Rome, Italy; ^3^Plastic and Reconstructive Surgery Unit, Magna Graecia University of Catanzaro, Catanzaro, Italy; ^4^Ophthalmology Unit, NESMOS Department, Sant'Andrea Hospital, Sapienza University of Rome, Rome, Italy

## Abstract

Reconstruction of the eyelids after skin cancer excision can be challenging. Surgical treatment options are multiple; deep anatomy knowledge of lamellar components is mandatory to choose the most adequate surgical planning. Eyelids' role in vision and social relationship is critical; both function and aesthetics are tough to restore. Using a flap provides a satisfying texture and colour match with adjacent tissues and ensures short contraction during healing; furthermore, grafts are sometimes necessary to achieve pleasing results. Hundreds of surgical techniques have been described aiming for eyelid reconstruction; in our paper, we want to provide for our audience the most reliable and useful procedures for subtotal and total eyelid reconstruction following NMSC full-thickness excision.

## 1. Introduction

Reconstruction of the eyelids is one of the most challenging areas in reconstructive plastic surgery. Perhaps no other region of the human body provides such a delicate interaction among anatomy, function, and aesthetics [1].

The primary goal in eyelid reconstruction is to restore a functional eyelid that protects the eye and permits normal vision. Furthermore, a normal tear film maintenance is another prerequisite.

The most important secondary objective is a normal appearance because of the critical importance of periocular region in social relationships. Surgical objectives comprise the following:

- Nonkeratinizing mucosal epithelium to line the inside of the reconstructed eyelid
- Firm connective tissue frame to provide support and shape which has to be posteriorly attached to the globe in all areas
- Adequate protractor muscle and supple, thin skin that permit normal eyelid movements
- Stable eyelid margin that prevents inappropriate turning

Surgical planning is made easier by conceptually dividing the eyelids into anterior and posterior lamellae. The anterior lamella is composed of the skin and the orbicularis muscle; the posterior lamella is composed of the conjunctiva, tarsus, and the eyelid retractors. For full-thickness defects, both lamellae usually require reconstruction. Unlike head and neck malignant melanoma, not always the whole facial aesthetic unit is to be replaced [2, 3].

Dealing with an eyelid defect, the surgeon should analyse the missing lamellar components and whether canthal support is compromised. Special attention should be paid to the integrity of the lacrimal apparatus when the resection involves the medial canthal region. The reconstructive plan will be determined mainly by the size of the defect and the status of the surrounding periorbital tissue. Single-stage procedures should be preferred. Microsurgical reconstruction, which is always more exploited in head and neck surgery [4], is not indicated in eyelid reconstruction.

Eyelid defects that span more than one-fourth of the eyelid width cannot be closed directly, with or without cantholysis, and require a more complicated reconstructive approach [5].

### 1.1. Direct Closure

An eyelid defect of 25% or less may be closed directly in most patients [6]. When combined with cantholysis, even a defect occupying up to 50% or more of the eyelid may be closed directly [7].

**Figure 1:**
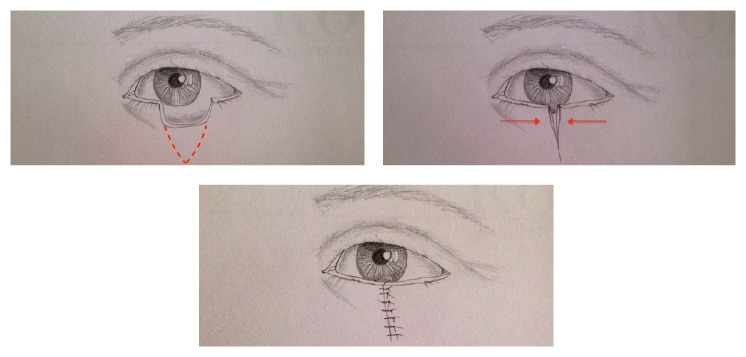
Direct closure for a defect of 25% or less of eyelid's width. Free-tension closure is achieved.

Excessive tension should be avoided because it can cause postoperative ptosis, especially in elderly patients [8]. An accurate gray line 7-0 silk suture is placed to evaluate the amount of tension and align the edges. When satisfactory position is achieved, the tarsal edges are approximated using interrupted 6-0 sutures. Then, muscle and skin layers are closed with interrupted 6-0 vicryl and 6-0 nylon, respectively (Figure 1). Pentagonal excision also offers reliable results [9].

## 2. Grafts in Eyelids Reconstruction

As an essential principle in plastic surgery, grafts should be used when there is a suitable vascular bed to enhance their survival.

Anterior lamellar defects can be reconstructed with a full-thickness skin graft [10]. Ideal donor sites include excess upper and lower eyelid skin and posterior auricular, preauricular, or supraclavicular skin [11]. Split thickness skin grafts should be avoided.

Tarsoconjunctival grafts are an excellent choice for posterior lamellar reconstruction, following the paradigm of replace like-with-like. They are harvested from the upper eyelid, leaving at least 3 to 4 mm of distal tarsus to avoid upper lid distortion. The donor site heals by secondary intention. Excellent results have been reported using tarsoconjunctival grafts for repairing defects of up to 75 percent of the eyelid length [12].

Furthermore, posterior lamella can be reconstructed using hard-palate mucoperiosteal grafts due to their ability to provide structural support and mucosal lining. They have been shown to produce reliable results; however, donor-site and recipient-site morbidity can be relevant [13, 14].

**Figure 2:**
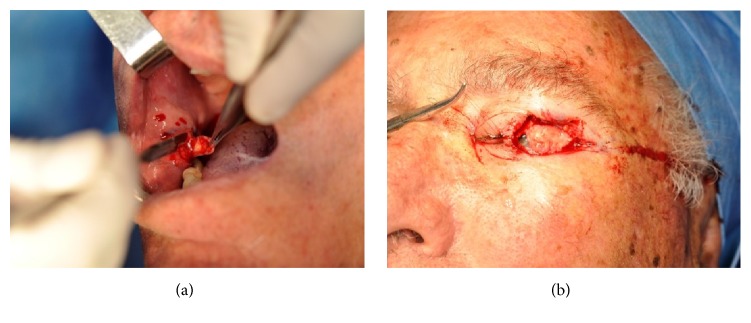
Oral mucosa graft for posterior lamella reconstruction. A graft of oral mucosa is harvested from the cheek (a). The graft is used for posterior lamella reconstruction (b).

Alternative options for posterior lamella reconstruction include nasal chondromucosa, auricular cartilage, and buccal mucosa, even if it does not provide a hard support for the anterior lamella (Figure 2) [15].

### 2.1. Composite Grafts

Composite grafts can be harvested from contralateral upper or lower eyelid to provide a replace like-with-like. The primary drawback of this reconstructive option is the donor-site morbidity.

Composite grafts of tarsus and conjunctiva can be employed for posterior lamellar reconstruction or, including skin, with interposed blood supply (muscle) for subtotal eyelid reconstruction.

When the lower eyelid full-thickness defect is not deep (5 to 10 mm in height) and if concomitant poor skin laxity does not allow raising a flap, the harvest of a full-thickness pentagon from the contralateral upper or lower eyelid can be performed, and the muscle has to be removed.

The graft is sustained by the orbicularis oculi blood supply that is undermined and sutured into the space left by the divided muscle. The graft edges are sutured to the recipient site. The donor site is repaired by direct closure.

## 3. Local Flaps for Subtotal and Total Upper Eyelid Reconstruction

Reconstruction of upper eyelid defect is very intricate, because, unlike the lower eyelid, there is not enough available tissue around it and preservation of function and contour is more demanding to achieve [16].

The levator muscle's function has to be respected; an inadequate dynamic function can compromise the visual axis of the patient; good eyelid closure is necessary to prevent exposure keratopathy due to lagophtalmos. For maintenance of corneal integrity and clear vision, upper eyelid must have 5 to 10 mm of movement and blink.

Many procedures have been reported for upper eyelid reconstruction; these techniques include the multiple composite eyelid grafts [17], chondromucosal flap [16] the Cutler-Beard flap from the lower eyelid [18], the inferiorly based tarsoconjunctival flap [19], the tarsoconjunctival rotational flap only for limited defect [20], the tarsoconjunctival horizontal advancement flap [21], and medial or temporal forehead flaps [22].

Some of them are two-stage technique; their main drawbacks are partial sacrifice of ipsilateral or contralateral normal eyelids, exposing the normal eyelids at risk for complications, or producing donor-site scarring. Furthermore, temporary occlusion of the eye (6 to 8 weeks) increases recovery time and enhance impediments for patients who are monocular or young enough to be at risk for deprivation amblyopia [23].

Here we report the techniques that, in our opinion, are the most reliable.

### 3.1. Horizontal V-Y Myotarsocutaneous Advancement Flap

The V-Y myotarsocutaneous flap is performed for upper eyelid defects up to 60% of eyelid width. It is designed on the lateral canthal region, pedicled on orbicularis oculi and extending at the crow's feet in a “V” fashion. The medial side of the flap corresponds to lateral margin of the defect; the superior edge follows the line of the superior palpebral fold. The flap height-to-width ratio can reach 1 : 4.

**Figure 3:**
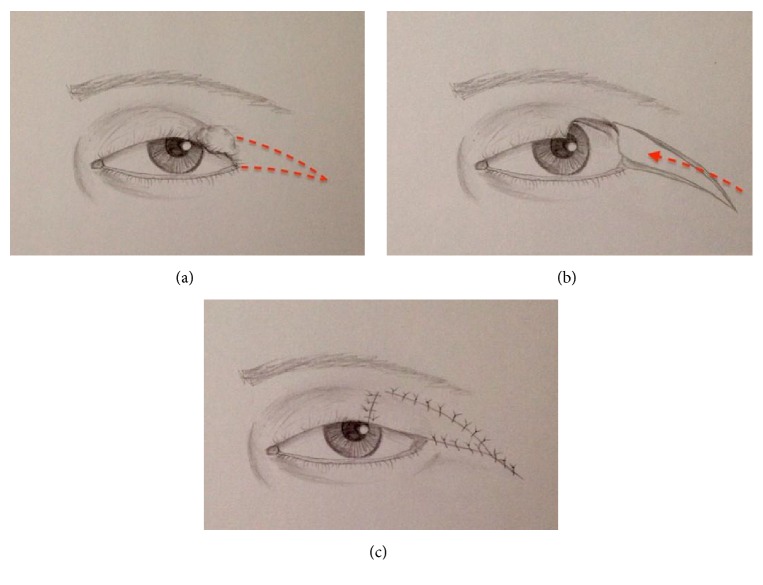
The V-Y myotarsocutaneous flap. Flap is designed on the lateral canthal region (a). Tumor excision, the medial side of the flap corresponds to lateral margin of the defect, and the superior edge follows the line of the superior palpebral fold (b). Flap advancement and donor-site closure in a V-Y fashion (c).

The incisions are performed until the orbicularis muscle in the upper eyelid, until the subcutaneous tissue in the cantal region. After eyelid eversion, an incision at the level of superior palpebral fold is made through the conjunctiva and tarsus. Then the flap is advanced with a gentle pull (Figure 3).

To increase flap's mobility it could be necessary to perform a lateral canthotomy [24].

#### 3.1.1. Anterior Lamella Reconstruction

When the reconstructive surgeon has to face a partial-thickness defect, anterior lamella, the use of orbicularis oculi myocutaneous flaps designed in various fashions has a greater aesthetic outcome compared with full-thickness skin grafts. Using a flap provides a better texture and colour match with adjacent tissues and ensure less contraction during healing. Lower eyelid reconstruction can be performed with reliable results using Blasius, Imre, or Tripier flap; upper eyelid reconstruction can be performed with Fricke flap. These transposition or rotation-advancement flaps can be combined with a tarsoconjunctival graft for posterior lamellar reconstruction in case of full-thickness defect.

**Figure 4:**
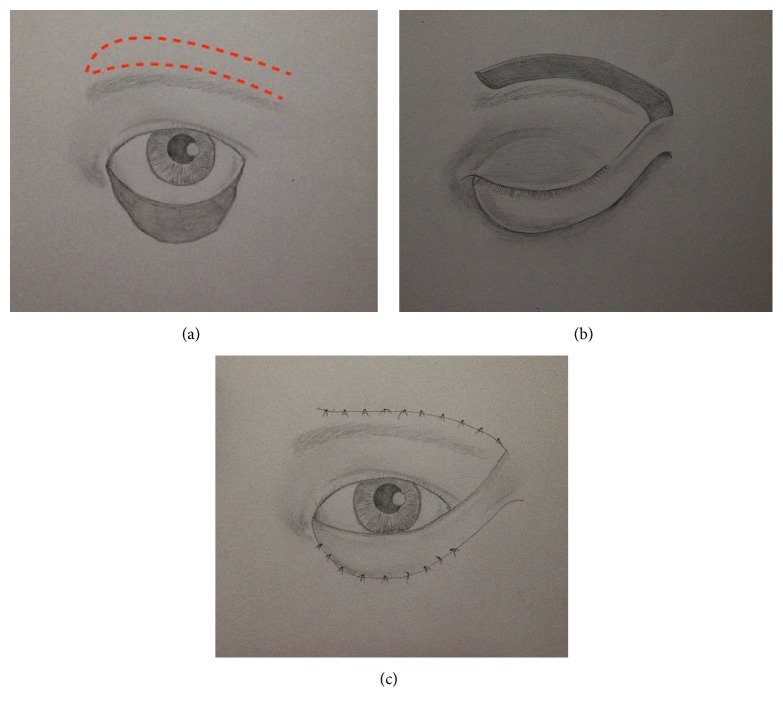
Fricke flap designed for total lower eyelid reconstruction (a). The transposition flap is raised and sutured (b). Donor site is closed (c).

**Figure 5:**
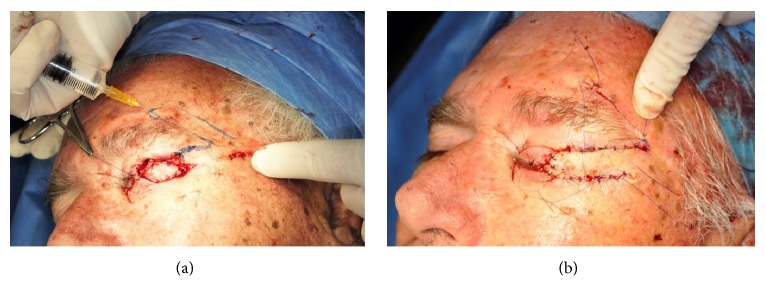
Fricke flap for subtotal upper eyelid reconstruction (anterior lamella). Intraoperative marking of Fricke flap, the posterior lamella has already been reconstructed using a buccal mucosa graft (a); the transposition flap and donor site are sutured (b).

*The Fricke Flap*. The Fricke flap is a cutaneous flap from the supraorbital area used for both upper and lower lid defects [25]. In the first stage, the length of the flap is marked out, based on the defect, just above the brow. The lateral aspect of the flap is kept as wide as possible and should be at least 1 : 4 for the base to length dimension of the flap. The flap is dissected along the plane between the subcutaneous tissue and the underlying muscles of the brow and lower forehead. Special attention should be paid to avoid the excessive thinning of the flap. Then, it is transposed to the eyelid defect. The wound closure is achieved with interrupted 5/0 vicryl sutures and nylon 6/0 suture. The donor-site wound is closed with interrupted subcutaneous 5/0 vicryl sutures and simple interrupted 6/0 nylon sutures are used for the skin closure (Figures 4 and 5).

It is useful in the monocular patients that the occlusion of the visual axis with techniques such as the Hughes or Cutler-Beard flaps would be unacceptable. The effectiveness and reliability of the technique are potentially enhanced when used in combination with rapid intraoperative tissue expansion [26, 27].

*Frontalis Muscle Flap*. Frontalis muscle flap is a reconstructive option for subtotal and total upper eyelid defect. It is elevated from the preexisting defect, thus avoiding an additional forehead scar. The posterior lamellar reconstruction is made by palatal mucoperiosteal graft.

After the posterior lamella is reconstructed, the levator palpebrae is reinserted and stitched to the periosteum of the graft.

The dissection is carried through the preexisting palpebral defect in a preseptal plane over the supraorbital rim. This dissection then reaches a subcutaneous plane over the frontalis muscle. A caudally pedicled frontalis muscle flap was then elevated and turned down to extend from the superior orbital rim to the reconstructed eyelid margin. The flap was tailored to match the defect without tension and sutured to the wound edges. A full-thickness skin graft is used to replace the upper eyelid skin.

The main advantage of this procedure is the absence of scar at the donor site [28].

#### 3.1.2. Posterior Lamella Reconstruction

*Chondromucosal Flap for Total and Subtotal Upper Eyelid Reconstruction*. Chondromucosal flap is a reconstructive option for the posterior lamella; the flap is designed along the lateral nasal wall and is based on the terminal branch of the ipsilateral dorsal nasal artery. It is an axial chondromucosal flap covered with a skin graft for the anterior lamella.

It is the preferred authors' technique in case of subtotal and total defects of the upper eyelid, even if it may be used for lower eyelid reconstruction as well.

The operative procedure starts with an incision at the border between the nasal sidewall and the cheek from the medial canthus to nasal ala. The flap is harvested in a subperiosteal plane from lateral to medial a bit beyond the midline of the nose and from the glabella to the lower margin of nasal bones.

**Figure 6:**
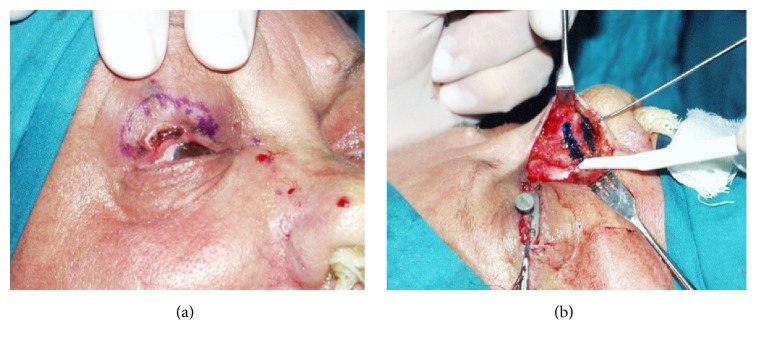
Chondromucosal flap. Preoperative picture, tumor excision is going to involve the whole width of upper eyelid (a). Intraoperative marking of chondromucosal flap, lateral nasal wall (b).

**Figure 7:**
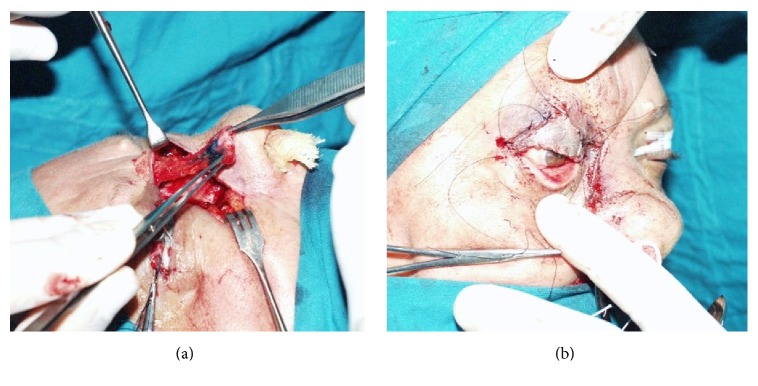
Chondromucosal flap. The flap is raised on his pedicle (a). The flap is covered with a full-thickness skin graft. (b).

**Figure 8:**
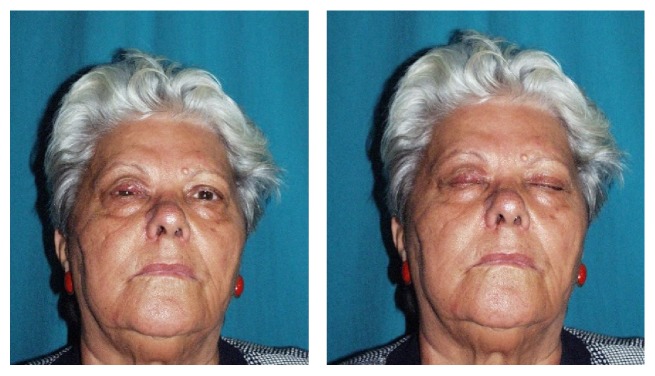
Chondromucosal flap. Postoperative view at 12 months.

Then the dissection is performed in the subcutaneous plane from lateral to medial and beyond the midline of the nose where it joins the subperiosteal plane. Distally the flap is harvested including the cranial portion of the upper lateral cartilage, then it is transposed to reconstruct the posterior lamella; flap mucosa is sutured to conjunctival margin and the levator stump is inserted into the cartilaginous portion. The anterior lamella is reconstructed with a skin graft harvested from the contralateral upper eyelid (Figures 6, 7, and 8). A blepharorrhaphy is performed to enhance skin graft take and avoid retraction of reconstructed eyelid. The donor-site defect is repaired with direct closure or left heal spontaneously [16].

*Cutler-Beard Flap*. The Cutler-Beard lid-sharing flap is a skin-muscle-conjunctiva flap harvested from the lower eyelid and advanced to cover upper eyelid defect up to 100% in width.

After tumor excision, the eyelid's stumps are brought toward each other with skin hooks to evaluate the width of the defect. A line is drawn on the lower eyelid parallel to the lid margin and 5 mm inferior to permit a fair blood supply at the lower eyelid margin. The flap has to be harvested 2 mm wider than the defect assessed and then vertical lines are drawn from medial and lateral end for 15 to 20 mm.

The flap is harvested along the lines and is undermined in suborbicularis plane to create a composite flap with conjunctiva and eyelid retractor system and then is advanced under the “bridge” of lower eyelid margin and stitched, respecting different layers, to upper eyelid defect.

The second stage of the procedure is performed 4 to 6 weeks after reconstruction. A horizontal incision is made 2 mm inferior to the new upper eyelid margin. The conjunctiva edge is sutured to the skin edge. At the donor-site, the inferior side of the bridge is freshened and sutured to the caudal part of the lower eyelid flap.

The main drawback of this procedure is the lack of a rigid posterior lamella which can lead to instability of the reconstructed eyelid; free tarsoconjunctival grafts or hard-palate mucosa grafts can be used to provide support to the flap (after flap inset) [18].

*Pedicled Lower Lid Sharing*. It was first described by Mustardé [29]. The flap is based on the central portion of the lower lid to reconstruct upper lid defect. The blood supply depends on the medial inferior marginal arcade because a lateral canthotomy is often required to allow direct closure of the donor site. The flap is rotated superiorly and the lateral lower lid tarsal plate is sutured to the medial upper eyelid tarsal plate. Generally, flap division is carried out after 6 weeks, although earlier flap division can be performed successfully.

*Sliding Tarsoconjunctival Flap*. The sliding tarsoconjunctival flap is a transposition flap based on adjacent conjunctival defects involving the medial or lateral upper eyelid [1].

The upper eyelid tarsus is incised horizontally 4 mm above the eyelid margin and the superior portion of the tarsus is then dissected from the orbicularis muscle and advanced horizontally along with its levator and Müller's muscle attachments. A tarsoconjunctival flap is raised and moved horizontally into the defect. It is then sutured in a side-to-side fashion to the lower portion of the residual upper lid tarsus using 5/0 Vicryl sutures and to a lateral or medial periosteal flap. A full-thickness skin graft or flap can be placed over the tarsoconjunctival flap for anterior lamellar reconstruction.

## 4. Local Flaps for Subtotal and Total Lower Eyelid Reconstruction

### 4.1. Anterior Lamella Reconstruction

#### 4.1.1. Tenzel Flap

Tenzel semicircular advancement flap is a useful technique in eyelid reconstruction both superior and inferior; it can be performed for full-thickness defects ranging from 30% to 70% in horizontal length of the eyelid; it is a single-stage reconstruction. The best results are achieved when there is availability of at least a little strip of full-thickness eyelid at the medial and lateral side of the defect. A periosteal flap or posterior lamellar graft is required in case of lateral tarsus absence.

*Surgical Technique for the Lower Eyelid Reconstruction*. Local anesthetic is administered. Starting at the lateral canthus a line is drawn with a semicircular pattern towards the lateral eyebrow. For the upper eyelid a mirror line is designed.

**Figure 9:**
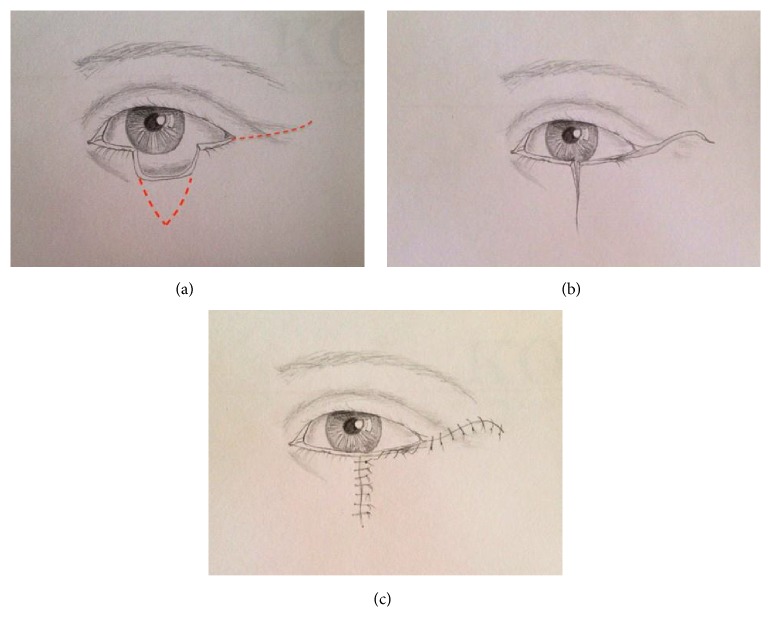
Tenzel flap. The tumor is excised. Starting at the lateral canthus a line is drawn with a semicircular pattern towards the lateral eyebrow (a). The lateral edge of the defect is advanced (b) and sutured (c) to the medial one.

Canthotomy is performed and the flap is dissected in a suborbicularis plane and completely undermined; then the lateral edge of the defect is advanced and sutured to the medial one (Figure 9).

The deep surface of the flap should be secured to the superolateral orbital rim periosteum to prevent eyelid sagging [30].

Furthermore, an enhanced eyelid contour can be obtained by sculpting a hinge periosteal flap from the lateral orbital rim; this is sutured to the inner side of the flap at the medial most extent as possible.

An alternative to periosteal flap for the posterior lamellar reconstruction is conjunctiva advancement from the lateral fornix, even though it provides a lesser optimal eyelid support and contour [31].

#### 4.1.2. The Nasojugal Flap

The nasojugal flap is based on the medial aspect of the lower eyelid and extended to the nasojugal fold. The nasojugal flap reconstruction is undertaken as a single-stage procedure. The medial aspect of the flap is kept as wide as possible and should at least respect the ratio of 4 : 1 for the base to length dimension of the flap. It is dissected along the subcutaneous plane. The flap is transposed to lie in the lower eyelid defect. The wound closure is achieved with interrupted 5/0 Vicryl sutures and interrupted 6/0 Nylon sutures for the skin layer. In a similar way, the donor-site wound is closed.

#### 4.1.3. Tripier Flap

It is based on excess upper lid skin and orbicularis to reconstruct defects of the lower lid [32]. The flap is marked out above the upper lid crease and transposed to the lower eyelid defect. Both donor-site and recipient site are sutured with 6/0 Nylon sutures.

#### 4.1.4. Rotation Cheek Flap (Mustardé Flap)

**Figure 10:**
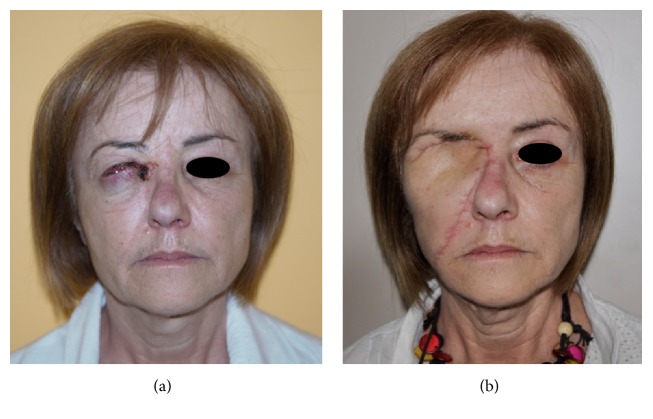
Mustardé flap. NMSC of upper and lower eyelid (a). Orbital exenteration and reconstruction with Mustardé flap were performed. Postoperative view at 2 months (b).

It is suitable for large lower lid defects [33, 34]. This flap is a good option for reconstruction of deep vertical defects and complete lower lid coverage in a single procedure [35]. A semicircular suborbicularis flap is designed at the lateral canthus and then extended laterally to the preauricular sulcus; it is elevated in a subcutaneous plane or sub-superficial musculoaponeurotic system for additional blood supply. A triangle of tissue is frequently excised inferior and medial to the defect for a better rotation and closure of the flap. Then, medial canthal fixation of the flap is suggested to avoid postoperative ectropion (Figure 10).

### 4.2. Posterior Lamella Reconstruction

#### 4.2.1. Hughes Flap

Hughes tarsoconjunctival flap advances the tarsal plate and conjunctiva from the ipsilateral upper eyelid to repair a defect in the lower eyelid (more than 60% in width) in a two-stage approach. The anterior lamella is reconstructed with a semicircular flap, a local myocutaneous flap, or a full-thickness skin graft.

The size of defect is evaluated by grasping both stumps of lower lid and carrying them together with skin hooks, and the width of tarsoconjunctival flap is drawn to be slightly shorter than the defect; this secures that there will be suitable horizontal tension of the eyelid. The most inferior 4 mm of tarsus within the upper eyelid has to be spared to provide an acceptable stability and contour of the donor-site.

After local anesthetic infiltration upper eyelid is everted, surgical marker is employed to draw a line parallel to the eyelid margin on the inner surface of the tarsus; the height is usually 4–6 mm at the highest point, then narrowing at the medial and lateral side. Conjunctiva and tarsus are then divided sparing the levator aponeurosis and dissection is continued until the flap can be advanced to cover the lower lid defect without excessive wound closure tension. The flap is sutured to tarsal stumps. Conjunctiva is advanced from the inferior fornix and secured to the lower border of the flap. Anterior lamella is reconstructed.

The Hughes flap causes temporary blindness due to obstruction until it is cut during second procedure after 14 days. The flap is divided into the planned new position of the lower eyelid.

This technique should be named as modified Hughes flap; it was quite different in Hughes first paper [36] but subsequently refined by Hughes himself and other authors; it is nowadays widely used with low rate of complications, the superior functional and esthetical outcome, and high patient satisfaction [37, 38].

## 5. Canthal Region

### 5.1. Glabellar Flap

The glabellar flap is a V-Y flap used for medial canthal reconstruction. The preoperative mark of the flap begins with the location of the apex of the V within the glabellar region. One arm of the V arises directly from the defect and passes superomedially toward the apex across the medial brow. The other arm arises from the apex at an angle to the first arm and passes inferiorly. The flap is elevated in a subcutaneous plane and advanced to reconstruct the lower eyelid. Donor site is sutured with 5 or 6/0 Vycril and 5/0 Nylon, whereas the deep surface of the flap is sutured to the periosteum with a 5/0 Vicryl suture to reform the concave contour of the medial canthus.

#### 5.1.1. Lacrimal Apparatus

Approximately 20% of eyelid malignancies occur in the medial canthus. It is a risky area because of lacrimal structures: the puncta, canaliculi, and the nasolacrimal duct. Partial or total loss of such structures is sometimes necessary to achieve a complete margin control. Even, when they are not directly violated by the scalpel for oncological reason, the postsurgical scarring and stenosis can easily impair the tear drainage system resulting in watery eye with blurred vision, intermittent or constant tearing (epiphora), and acute or chronic dacryocystitis.

When the punctum and partial proximal canaliculus are involved, a simple mono- or bicanalicular silicone lacrimal intubation is the procedure of choice leading to a good rate of success with adequate lacrimal drainage [32, 39].

When faced with a complete loss of canaliculus, a reconstruction with buccal mucosa enveloping silicone stent placed between anterior and posterior lamella may be necessary. Such a challenging reconstruction often gives poor and disappointing results; epiphora can be prevented performing a conjunctivodacryocystorhinostomy with Jones tubes, a permanent fistula between the medial fornix and the nasal cavity.

Dacryocystorhinostomy is indicated when the nasolacrimal duct is partially or totally obstructed and the canaliculi are patent.

The approach to the procedure has changed during last decades, from an external to an endonasal one that shows more favourable results and does not require any damage to structure such as orbicularis muscle and medial palpebral ligament.

The mucosa of the nasal sidewall is incised and elevated. The procedure is endoscopic; all the bone of the lacrimal fossa has to be removed to allow the lacrimal sac to be flatter on nasal lacrimal wall. The lacrimal system is then intubated with a bicanalicular silicon stent. The success rates are between 75% and 97% [40].

## 6. Conclusion

Eyelid reconstruction could be a challenging procedure for a surgeon, due to its particular anatomy and function. The analysis of the eyelid defect and a correct preoperative plan is mandatory. Each component of upper and lower eyelids as the anterior lamella, posterior lamella, and tarsoligamentous sling should be investigated and, if required, reconstructed with the appropriate technique, ensuring an adequate vascular supply. The principle of when to use a graft, direct closure, a distant flap, or lid-sharing procedures is the key point of successful reconstruction. A single-stage reconstruction should be the goal without compromising the aesthetic and functional results.
